# Retraction: Novel microalgae strains from selected lower Himalayan aquatic habitats as potential sources of green products

**DOI:** 10.1371/journal.pone.0274857

**Published:** 2022-09-30

**Authors:** The PLOS ONE Editors

The *PLOS ONE* Editors retract this article [1] because it was identified as one of a series of submissions for which we have concerns about authorship, competing interests, and peer review. We regret that the issues were not addressed prior to the article’s publication.

IZ, QM, UI, MI, FH, AI, and RN did not agree with the retraction. ABI and AP either did not respond directly or could not be reached.
